# Nicotinamide N-methyltransferase expression and its association with phospho-Akt, p53 expression, and survival in high-grade endometrial cancer

**DOI:** 10.3906/sag-1907-166

**Published:** 2019-10-24

**Authors:** Serra AKAR, İsmail HARMANKAYA, Serdar UĞRAŞ, Çetin ÇELİK

**Affiliations:** 1 Division of Gynecologic Oncology, Department of Obstetrics and Gynecology, School of Medicine, Selçuk University, Konya Turkey; 2 Department of Pathology, School of Medicine, Selçuk University, Konya Turkey

**Keywords:** Nicotinamide N-methyltransferase, type II endometrial cancer, Akt, p53, disease-specific survival

## Abstract

**Background/aim:**

Nicotinamide N-methyltransferase (NNMT) is an enzyme that is overexpressed in malignancies. NNMT expression has not been previously studied in endometrial cancer (EC). Increased phospho-Akt (pAkt) levels in response to NNMT overexpression have been reported in in vitro studies of different cancer types. We assayed NNMT expression in primary and metastatic high-grade EC and investigated the relationship of NNMT with p53, pAkt, and survival.

**Materials and methods:**

NNMT, pAkt, and p53 expressions were assayed in 100 tissue samples of benign endometria, primary EC, and metastatic EC by immunohistochemistry.

**Results:**

The NNMT immunoreactivity score was significantly higher in primary high-grade EC than benign endometrial tissue (P = 0.001). NNMT expression in metastatic tissue was significantly higher than in primary cancer (P < 0.001). Metastatic stromal NNMT expression was significantly higher than that of the adjacent tumor and stroma adjacent to the primary tumor. p53 expression in the primary tumor showed a significant positive correlation with omental NNMT and pAkt expression. NNMT expression was also correlated with pAkt expression in metastatic tissue. NNMT overexpression in metastatic tissue was associated with decreased survival (P = 0.039).

**Conclusion:**

This study suggests that NNMT may promote cancer progression and that NNMT overexpression is associated with aberrant p53 expression, pAkt, and poor survival. NNMT’s role in cancer progression could make it a target of EC therapy.

## 1. Introduction

Endometrial adenocarcinoma is the most frequent form of uterine cancer, which is the leading gynecological cancer in countries with adequate cervical cancer prevention programs [1]. High-grade endometrial cancer (EC) comprises a histological subtype of tumors that are more aggressive in nature than low-grade tumors and include serous, clear cell, grade 3 endometrioid adenocarcinomas, undifferentiated carcinomas, and carcinosarcomas. Although high-grade tumors make up only 10%–20% of EC, they are responsible for a disproportionate 40% of mortality [2,3]. Unlike patients with type I EC, patients with type II EC often present at advanced stages with metastases. 

Nicotinamide N-methyltransferase (NNMT) is a cytosolic enzyme that is overexpressed in a variety of human malignancies including lung cancer [4], glioblastoma [5], and gastric [6], pancreatic [7], and colorectal [8] cancers. It is consistently associated with tumor aggressiveness, progression, invasion, and metastasis [5,9,10] and its inhibition may provide therapeutic benefit [11]. NNMT overexpression has been shown to lead to the phosphorylation and thereby the activation of the oncogenic Akt, also known as protein kinase B. Akt is a serine/threonine kinase known to inhibit apoptosis [5,9,12]. 

This is the first study to investigate NNMT expression in primary and metastatic EC. We sought to assay NNMT expression in benign endometrial tissue, high-grade endometrial neoplasms, and matched metastatic omental tissue. Furthermore, we attempted to investigate the relationship of NNMT with p53 and phospho-Akt (pAkt) in metastatic EC. Additionally, the association of disease-specific survival (DSS) with NNMT expression was analyzed. 

## 2. Materials and methods

### 2.1. Patient samples

A total of 100 formalin-fixed paraffin-embedded tissue blocks from patients who received surgical treatment for endometrial cancer in a single institution between 2009 and 2018 were included in the study. Thirty patients were diagnosed with stage III high-grade EC (serous, clear cell, and grade 3 endometrioid endometrial adenocarcinoma) based on the World Health Organization criteria [13] and the International Federation of Gynecology and Obstetrics (FIGO) classification for stage and grade [14,15]. Their primary tumor and matched metastatic omental tissue samples were obtained as a part of debulking surgery and were analyzed for the study. Patients underwent debulking surgery with subsequent administration of appropriate adjuvant therapy. Benign omental tissues without metastatic involvement from 20 patients were collected for the study. These benign omental tissues were obtained from separate sets of patients who underwent surgeries for benign indications such as benign ovarian cysts that caused adhesions extending between the omentum and the adnexa or benign hysterectomies in patients with repeated laparotomies that required a partial omentectomy due to adhesions. Paraffin blocks of benign endometrial tissue of 20 patients who were diagnosed with benign endometrial conditions following a hysterectomy were used for comparison. NNMT expression was analyzed in all tissues while pAkt expression was analyzed in benign omental and metastatic omental tissues only. Patients with a previous history of malignancy or receipt of chemotherapy or radiotherapy were excluded from the study. The study was approved by the Institutional Ethics Committee of Selçuk University Medical School (Konya, Turkey). 

Data on age, histopathological information including histomorphological diagnosis, stage, and p53 immunoreactivity of the primary tumor were retrieved and reviewed from patient records. Ancillary p53 testing by anti-p53 antibody (DO-7) (#M7001, Dako, Glostrup, Denmark) had been used to detect aberrant p53 expression in the endometrial tumor during initial histopathological analysis following surgery. For this study, p53 immunoreactivity was grouped based on the WHO classification [13], whereby >70% diffuse nuclear p53 positivity due to a missense mutation or complete absence of staining (<5%) due to a nonsense mutation is indicative of aberrant p53. Other focal (5%–70%) patterns of p53 positivity were classified as normal p53 staining. DSS, as the interval from the date of diagnosis to time of death due to disease, was calculated from follow-up records and the National Death Registry, last checked on 12 April 2019. 

### 2.2. Immunohistochemistry

The tissue blocks were sliced into 4-μm sections, after which the sections were deparaffinized and rehydrated. Heat-induced antigen retrieval was performed with 10 mM sodium citrate buffer (pH 6.0). Endogenous peroxidase was quenched with 3% hydrogen peroxide for 15 min. Monoclonal mouse antihuman antibody for NNMT (dilution 1:400, Novus Biologicals, USA, NBP2-00537) and pAkt (ser473) (dilution 1:100, Cell Signaling Technology, USA, D9E) was applied to detect protein expression. Following the addition of the secondary antibody, diaminobenzidine was applied for 3 min for the chromogenic reaction. Hematoxylin-eosin stain was used for counterstaining. 

Immunoreactivity for each slide was evaluated by two experienced blinded pathologists without knowledge of the clinicopathological characteristics of the patients. The overall staining score for the interpretation of NNMT and pAkt immunoreactivity was based on the percentage of positive cells multiplied by their corresponding staining intensity obtained under a microscope at 400×. The percentage of positive cells was calculated from the examination of 1000 cells under 40× view. The staining intensity was classified as follows: 0 (no staining), 1 (weak staining), 2 (moderate staining), 3 (strong staining), and 4 (very strong staining). The overall staining score was calculated from the addition of all the products obtained by multiplication of different staining intensities and their corresponding percentages. As such, the overall staining score had a minimum value of 0 (no staining) and a maximum value of 400 (100% of cells with staining intensity of 4). NNMT expression was further analyzed within the tumor and its surrounding stroma separately for both primary and metastatic tumors. 

### 2.3. Statistical analysis

The Mann–Whitney U test was used to compare NNMT expression scores between unrelated samples. The Wilcoxon signed-rank test was used to compare NNMT scores between matched primary and metastatic cancer as well as between the tumor and its adjacent stroma in primary and metastatic tumors. Correlation of NNMT expression with pAkt and p53 expression was obtained using Spearman’s rank-order correlation coefficient. The quantitative staining score data for NNMT and pAkt expression were converted into dichotic data of low and high NNMT and pAkt expression, respectively, by cut-off and sensitivity values calculated using ROC analyses. NNMT and pAkt scores below and above the cut-off values were considered to indicate low and high expression, respectively. Kaplan–Meier analysis was used to investigate the potential association between low and high NNMT expression and survival outcomes and the log-rank test was used to determine statistical significance. Variables P < 0.20 in univariate analyses were included in multivariate analysis (Cox proportional hazards model). P < 0.05 was considered statistically significant. All statistical analyses were performed using SPSS 24.0 (IBM Corp., Armonk, NY, USA). 

## 3. Results

The average age of patients with high-grade endometrial cancer and benign endometria was 63.9 ± 12.3 (34–82) and 50.0 ± 8.0, respectively. Histological subtypes included 15 (50.0%) high-grade uterine serous, 13 (43.3%) high-grade (grade 3) uterine endometrioid, and 2 (6.7%) uterine clear cell adenocarcinoma. p53 staining was done for 24 (80%) patients as part of the initial pathological examination. Nine (37.5%) of the patients had normal p53 staining while 15 (62.5%) patients had aberrant p53 staining patterns. Aberrant p53 staining was seen in 93% of patients with high-grade uterine serous carcinoma, 15% of patients with high-grade endometrioid uterine carcinoma, and 100% of patients with clear cell uterine adenocarcinoma. 

**Figure 1:**
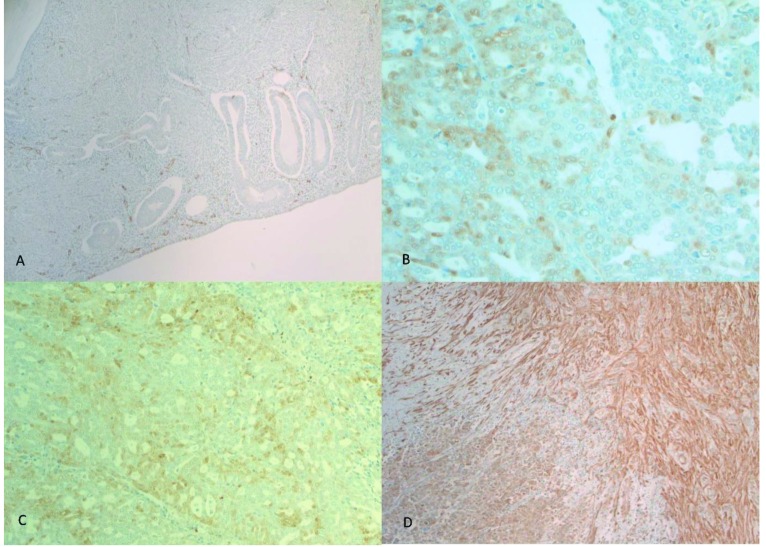
A) Benign proliferative endometrium with minimal NNMT expression mainly within vessels walls (original magnification 100×). B) Primary high-grade endometrial carcinoma showing NNMT immunoreactivity that was classified as “low” NNMT expression (original magnification 200×). C) Primary high-grade endometrial carcinoma showing NNMT immunoreactivity that was classified as “low” NNMT expression (original magnification 400×). D) Primary high-grade endometrial carcinoma showing predominantly cytoplasmic diffuse NNMT immunoreactivity that was classified as “high” NNMT expression (original magnification 100×).

**Figure 2:**
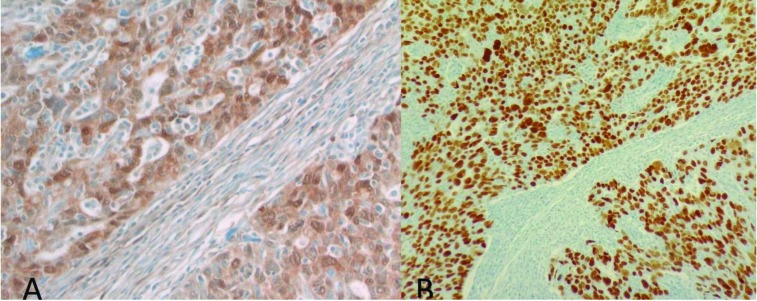
A). Primary tumor and stroma showing high NNMT expression (original magnification 400×). B) Diffuse strong nuclear p53 expression within the tumor cells of the same patient (original magnification 200×).

**Figure 3:**
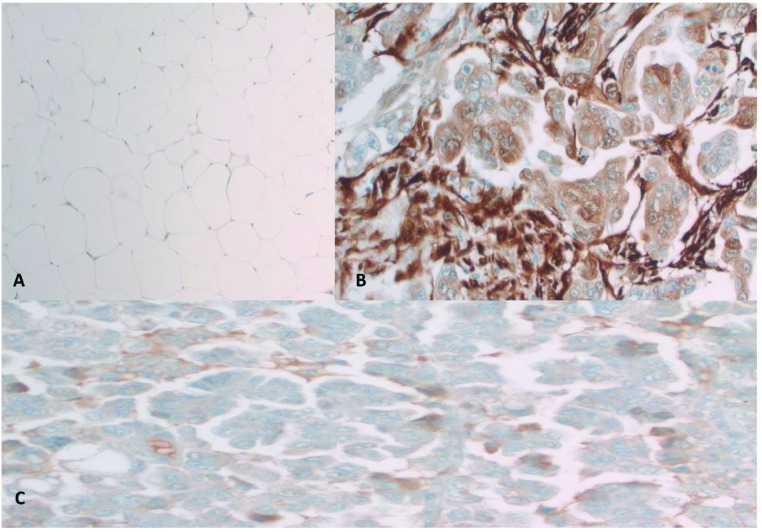
A) Benign omentum without NNMT immunoreactivity (original magnification 200×). B) Metastatic high-grade endometrial carcinoma in the omentum with higher expression of NNMT in stroma compared to tumor (original magnification 400×). C) pAkt expression in omental metastatic tumor.

NNMT immunoreactivity was not detected within benign endometrial stroma (<10%). Of note, blood vessels within benign endometrial stroma demonstrated NNMT immunoreactivity. Benign omental tissue did not display significant NNMT immunoreactivity. NNMT immunoreactivity was seen in 52.4%, 65.4%, 90%, and 90% of patients within the endometrial tumor, stroma adjacent to the tumor, omental metastatic tumor, and stroma adjacent to the metastatic omental tumor, respectively. The frequency of NNMT immunoreactivity in tumoral and stromal tissue in endometrial cancer was significantly higher than in benign endometrium (P < 0.001). The frequency of NNMT immunoreactivity was significantly higher in metastatic tumors compared to the primary tumors (P = 0.002). The frequency of NNMT immunoreactivity was significantly higher in metastatic stroma compared to primary tumor stroma (P = 0.027). NNMT immunoreactivity was mostly cytoplasmic but included nuclear reactivity as well. Illustrative samples of NNMT and pAkt immunoreactivity are presented in Figures 1-3.

Comparison of NNMT expression among benign endometrial tissue, primary EC, metastatic EC, and benign omental tissue is given in Table 1. The NNMT expression score was significantly higher in primary high-grade EC than benign endometrial tissue (P = 0.001). NNMT expression in metastatic omental tissue was significantly higher than in primary cancer tissue (P < 0.001). Of note, NNMT expression was significantly higher in the stroma adjacent to the metastatic tumor than the stroma adjacent to the primary tumor (P = 0.003) (Table 2). NNMT expression was significantly higher in the stroma than in its adjacent tumor in metastatic tissue (P =0.045) (Table 2). 

**Table 1:** Comparison of NNMT expression among benign, primary, and metastatic cancer tissues.

	Benignendometrium	Benignomentum	PrimaryEC	Metastatic EC	P
NNMT score (overall)	14.9 ± 10.1	25.8 ± 26.8	64.8 ± 71.7	140.3 ± 117.6	<0.001*
N	20	20	30	30	

*Benign endometrium vs. primary tumor, P = 0.001 (Mann–Whitney U test). Primary tumor vs. metastatic omental tumor, P < 0.001 (Wilcoxon signed rank test). Metastatic omental tumor vs. benign omental tissue, P <0.001 (Mann–Whitney U test).

NNMT: Nicotinamide N-methyltransferase, EC: endometrial cancer.

**Table 2:** Comparison of NNMT expression between primary and metastatic tumor and stroma in high-grade endometrial carcinoma.

	N	NNMT score	P
Primary tumor	30	59.0 ± 92.9 (0–270)	0.83
Metastatic tumor	30	72.5 ± 105.5 (0–360)	
Stroma adjacent to primary tumor	30	64.8 ± 71.7 (0–260)	0.003
Stroma adjacent to metastatic tumor	30	203.2 ± 96.5 (40–360)	
Primary tumor	30	59.0 ± 92.9 (0–270)	0.89
Stroma adjacent to primary tumor	30	64.8 ± 71.7 (0–260)	
Metastatic tumor	30	72.5 ± 105.5 (0–360)	0.045
Stroma adjacent to metastatic tumor	30	203.2 ± 96.5 (40–360)	

NNMT: Nicotinamide N-methyltransferase.

The mean omental stromal NNMT score was significantly higher in patients with aberrant p53 staining than patients with normal p53 staining in the primary tumor (P = 0.048) (Table 3). p53 expression in the primary tumor showed a significant positive correlation with omental stromal NNMT expression (r = 0.74, P = 0.023).

**Table 3:** Comparison of metastatic NNMT and pAkt scores between normal and aberrant p53 staining patterns in the primary tumor.

	N	Omental NNMT score	P	Omental pAkt score	P
Normal p53*	9	55.0 ± 80.8 (0–45)	0.048	6.2 ± 15.3	0.003
Aberrant p53º	15	204.3 ± 130.2 (0–360)		44.3 ± 14.8	

NNMT: Nicotinamide N-methyltransferase, pAkt: phosphorylated Akt.*p53 positivity in 5%–70% of cells; ºnuclear p53 positivity in <5% or >70% of cells.

pAkt score was significantly higher in metastatic omental tissue than omental tissue without metastatic involvement (40.5 ± 16.1 vs. 5.0 ± 14.1, respectively; P = 0.02). pAkt expression in omental tissue showed a strong degree of positive correlation with NNMT expression in metastatic stromal omental tissue (r = 0.801, P = 0.017). The mean pAkt score in the omentum was significantly higher in patients with aberrant p53 in the primary tumor than patients with normal p53 staining in the primary tumor (P = 0.024) (Table 3). p53 expression in the primary tumor showed a significant positive correlation with omental pAkt expression (r = 0.85, P = 0.004). 

**Figure 4:**
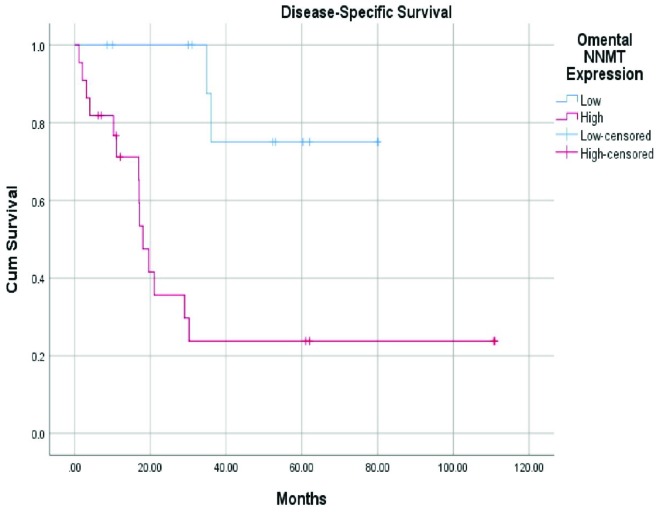
Disease-specific survival in patients with low and high metastatic NNMT expression (P = 0.039).

A score of 80 with 85% sensitivity and 80% specificity was found as the significant cut-off value for low and high metastatic NNMT stromal expression. The mean DSS for patients with low and high metastatic NNMT stromal expression was 68.8 months (95% CI: 45.8–88.0) and 37.8 months (95% CI: 13.4–20.6), respectively (P = 0.003) (Table 4; Figure 4). The mean DSS for patients with low and high pAkt expression was 68.9 months (95% CI: 42.1–96.0) and 2.1 months (95% CI: 0.23–3.9), respectively (P = 0.044) (Table 4). The mean DSS for patients with normal and aberrant p53 expression was 54.7 months (95% CI: 26.8–82.6) and 28.3 months (95% CI: 14.6–41.9), respectively (P = 0.23) (Table 3). NNMT retained its prognostic significance as an independent variable in multivariate survival analyses (P = 0.046) (Table 4). 

**Table 4:** Univariate and multivariate survival analyses of stage III type II endometrial cancer patients according to age, NNMT, pAkt score, and p53 expression pattern.

			Univariate analysis		Multivariateanalysis	
Parameter	Category	No. of cases	DSS		Hazard ratio(95% CI)	
			No. of events	P		P
Age	<60	18	6	0.57			≥60	12	8
	NNMT	Low	11				3	0.039	4.9 (1.0-13.5)	0.046	High	19	11
pAkt		Low	26	10	0.044	1.3 (0.9-3.4)	0.65			
	High	4	4				
p53	Normal	9	2	0.23		
	Aberrant	15	9			

DSS: Disease-specific survival, NNMT score: NNMT expression score in omental metastases of type II endometrial carcinoma, pAkt score: phospho-Akt expression score in omental metastases of type II endometrial carcinoma.

## 4. Discussion

To our knowledge, NNMT expression has not been studied in uterine cancer and high-grade EC thus far. The expression of NNMT was found to be higher in primary high-grade EC than benign endometrial tissue. Moreover, NNMT expression was significantly higher in metastatic EC than the matched primary tumor. Metastatic stromal NNMT expression was significantly higher than that of its adjacent tumor and stroma adjacent to the primary tumor. NNMT overexpression in metastatic tissue was associated with decreased survival. Furthermore, NNMT expression correlated with pAkt expression in metastatic tumor tissue and metastatic NNMT and pAkt expression correlated with p53 expression in the primary tumor. 

Although the majority of endometrial carcinomas are endometrioid and have a favorable prognosis, high-grade EC accounts for a substantial portion of mortality due to endometrial cancer [2,3]. NNMT is a metabolic protein shown to be involved in cancer dissemination, treatment failure, and recurrence [5,6]. Elevated NNMT levels contribute to tumorigenesis through DNA hypomethylation, which alters the expression level of a tumor suppressor protein, protein phosphatase 2A (PP2A) [5,9]. Decreased PP2A levels lead to phosphorylation (activation) of the oncoprotein Akt, which is a serine/threonine kinase also known as protein kinase B. Akt is an important component of the phosphatidylinositol 3-kinase (PI3K) signaling pathway. Mutations in the components of this pathway have been linked to tumorigenesis. Certain studies have shown NNMT overexpression to lead to an increase in the phosphorylated or activated form of Akt, which is an antiapoptotic protein associated with tumorigenesis [5,9,12]. In line with these in vitro studies, NNMT expression and pAkt expression showed a strong degree of positive correlation within metastatic high-grade EC. Aberrant p53 expression was significantly associated with higher expression of NNMT and pAkt. 

The progressive increase in NNMT expression from benign endometria to primary EC and finally metastatic EC suggests that NNMT plays a role in cancer development and metastasis. NNMT overexpression is critical in tumor metastasis via its induction of epithelial-to-mesenchymal transition (EMT), which is the transformation of epithelial cells into mesenchymal-like cells and is necessary for invasion and metastasis [16,17]. Transforming growth factor-β1 (TGF-β1), a key component of EMT, is shown to be elevated due to NNMT overexpression [16,17]. 

The stromal component of metastatic EC displayed higher NNMT expression than its matched adjacent tumoral component and primary tumor stroma. These findings indicate that NNMT may contribute to the desmoplastic tumor reaction, which is increased fibrous tissue surrounding the tumor and is associated with cancer progression and treatment failure [18]. In high-grade serous carcinoma of the ovary, the desmoplastic or mesenchymal type of cancer, categorized as such based on molecular profiling of stromal fibroblasts, has been associated with the worst survival [19]. Growth factors including TGF-β1 were differentially expressed between normal ovarian tissue and stroma around ovarian carcinoma [20]. Specifically, in endometrial cancer, cancer-associated fibroblasts (CAFs) have been shown to induce EMT and promote invasion and migration [21]. NNMT was recently found to be an important regulator of CAF activity and tumor progression in the stroma of ovarian cancer [11]. 

The association of metastatic NNMT overexpression with lower DSS is in keeping with results from earlier studies reporting NNMT overexpression in the primary tumor to be a poor prognostic factor in gastric cancer [6], pancreatic cancer [7], and hepatocellular carcinoma [22]. NNMT has been associated with EMT and cancer stem cells, which are recognized to play a role in metastasis, resistance to treatment, and recurrence [16,17,23–25]. 

Aberrant p53 expression in the primary tumor exhibited a strong association with NNMT and pAkt expression in the metastatic tumor. As with NNMT, p53 is shown to exert its effects through epigenetic changes involving histone methylation and acetylation [26]. Aberrant NNMT and p53 expression may be related and NNMT expression may increase following TP53 mutations. 

The major limitation of this study was the lack of NNMT immunoreactivity analysis of low-grade endometrial cancer samples. Additionally, no comparison of NNMT expression was made between primary tumors in patients with and without metastases. The small sample size is obviously a shortcoming of the study; however, it may lead to studies with larger sample size and those involving gene expression analyses of NNMT overexpression and p53 mutations. Another shortcoming of the study was its retrospective nature. Since this was a retrospective study, of the 30 patients included in the study, p53 immunoreactivity was available for only 24 patients as part of the diagnostic work-up in the initial pathological analysis. 

This study suggests that NNMT may promote cancer progression through desmoplasia and that NNMT overexpression in metastatic tissue is associated with poor survival. NNMT overexpression may be related to TP53 mutations and exploration of this potential relationship may reveal why aberrations of both proteins are associated with aggressive tumor behavior. NNMT’s role in cancer progression could make it a critical target of high-grade EC therapy.

## Acknowledgment

This study was funded by the Selçuk University Scientific Research Endorsement (#18401135).
